# Antibiotic Therapy in Acute Infectious Diarrhea and *Clostridioides difficile* Infection: Clinical Indications, Antimicrobial Stewardship, and Evidence Gaps

**DOI:** 10.3390/antibiotics15100957

**Published:** 2026-09-27

**Authors:** Roberta Rotondo, Piera Cicchetti, Serena Olivelli, Chiara Soriani, Susanna Esposito

**Affiliations:** Pediatric Clinic, Department of Medicine and Surgery, University of Parma, 43126 Parma, Italy; roberta.rotondo@unipr.it (R.R.); piera.cicchetti@unipr.it (P.C.); serena.olivelli@unipr.it (S.O.); chiara.soriani@unipr.it (C.S.)

**Keywords:** gastroenteritis, infectious diarrhea, antibiotic therapy, antimicrobial stewardship, antimicrobial resistance, precision medicine

## Abstract

Background: Most acute gastrointestinal infections are self-limiting, but antibiotics remain essential for selected patients with severe, invasive, or high-risk disease. This narrative review examines when antibiotics should be started or withheld in adults and children with suspected or confirmed bacterial gastrointestinal infection, which regimens should be selected, and how treatment should be reassessed after diagnostic and susceptibility results become available. Methods: PubMed and Scopus were searched for relevant English-language evidence published from 1 January 2014 through 28 August 2026. Google Scholar was used for supplementary citation tracking, and landmark earlier studies and major international guidelines were also considered. The search informed a narrative synthesis and was not conducted as a systematic review. Sixty-one sources were included. Results: The evidence base is uneven. Recommendations concerning rehydration, avoidance of antibiotics in Shiga toxin-producing *Escherichia coli* infection, and treatment of *Clostridioides difficile* infection (CDI) are comparatively consistent. By contrast, treatment thresholds and durations for several enteric infections rely largely on observational evidence, indirect evidence, or expert consensus. Antibiotics should generally be withheld in uncomplicated watery diarrhea and used selectively for dysentery, severe travelers’ diarrhea, cholera, enteric fever, invasive salmonellosis, and CDI. Regimen selection should reflect host risk, illness severity, travel exposure, and local susceptibility data. Therapy should be narrowed or discontinued after diagnostic clarification whenever appropriate. Conclusions: The principal stewardship priorities are appropriate patient selection, culture and susceptibility testing when the results may change management, and use of the shortest effective regimen supported by the available evidence.

## 1. Introduction

Acute gastrointestinal infections are common, but only a minority require antibacterial treatment. Rehydration and supportive care remain the foundation of management, whereas unnecessary antibiotics expose patients to adverse effects, disrupt the intestinal microbiota, increase the risk of *Clostridioides difficile* infection (CDI), and select antimicrobial resistance [1,2,3,4,5,6].

The practical difficulty is not simply identifying a possible pathogen. Clinicians must decide whether the expected benefit of immediate treatment exceeds the risks of unnecessary exposure, whether diagnostic results justify targeted therapy, and what duration is sufficient. This balance changes with disease severity, dysentery or sepsis, age, immune status, comorbidity, pregnancy, travel or outbreak exposure, and local susceptibility patterns [4,6,7,8].

Several high-impact exceptions frame this decision. Antibiotics are usually unnecessary in uncomplicated non-typhoidal salmonellosis and campylobacteriosis, but may be indicated in severe disease or in patients at increased risk of invasion. Treatment is generally indicated for enteric fever, severe shigellosis, severe travelers’ diarrhea, cholera with severe dehydration, and symptomatic CDI. By contrast, antibiotics should be avoided in suspected or confirmed Shiga toxin-producing *Escherichia coli* (STEC) infection because exposure may increase the risk of hemolytic uremic syndrome [5,6,7,9].

This narrative review examines a focused clinical question: in adults and children with suspected or confirmed acute bacterial diarrhea or CDI, when should antibiotics be started or withheld, which pathogen-directed regimens are supported by current evidence, and how should treatment be reassessed as diagnostic and susceptibility information becomes available? The review is narrative, was not designed as a systematic evidence synthesis, and does not follow PRISMA reporting requirements. It focuses on bacterial gastrointestinal infections and CDI. Viral and parasitic etiologies are not reviewed in detail, except where their recognition supports withholding antibacterial treatment. *Helicobacter pylori* infection, intra-abdominal infection, diverticulitis, and biliary infection are excluded because they have distinct diagnostic and therapeutic frameworks. Prevention and emerging technologies are considered only when evidence links them directly to antibiotic initiation, selection, de-escalation, or prevention of recurrent antibiotic-treated disease.

## 2. Methods

This narrative review addressed the following clinical question: in adults and children with suspected or confirmed bacterial gastrointestinal infection, which clinical and microbiological findings justify starting, withholding, narrowing, or stopping antibiotics, and which regimens and durations are supported for the major treatable syndromes? The review was intended to provide a clinically oriented critical synthesis rather than a systematic review or meta-analysis.

PubMed and Scopus were searched for English-language publications from 1 January 2014 through 28 August 2026. Google Scholar was used for supplementary citation tracking. The complete PubMed strategy was: ((“infectious diarrhea” OR gastroenteritis OR “travelers’ diarrhea” OR *Salmonella* OR *Shigella* OR *Campylobacter* OR “diarrheagenic *Escherichia coli*” OR cholera OR “*Clostridioides difficile*”) AND (antibiotic OR “antimicrobial treatment” OR resistance OR stewardship OR “rapid diagnostic” OR “microbiota therapy”)).

The complete Scopus strategy was: TITLE-ABS-KEY ((“infectious diarrhea” OR gastroenteritis OR “travelers’ diarrhea” OR *Salmonella* OR *Shigella* OR *Campylobacter* OR “diarrheagenic *Escherichia coli*” OR cholera OR “*Clostridioides difficile*”) AND (antibiotic OR “antimicrobial treatment” OR resistance OR stewardship OR “rapid diagnostic” OR “microbiota therapy”)). Reference lists of eligible guidelines and reviews were screened, and landmark earlier studies were retained when they remained central to current recommendations.

Two authors independently reviewed titles, abstracts, and full texts for clinical relevance. Disagreements were resolved through discussion with the senior author. Eligible sources included clinical practice guidelines, systematic reviews, randomized trials, comparative observational studies, resistance-surveillance reports, official product information, and public-health guidance addressing treatment indications, agent selection, duration, de-escalation, recurrence, or clinically relevant harms. Non-English publications, isolated case reports, unsupported opinion pieces, and reports without sufficient methodological detail or direct clinical relevance were excluded.

The search was iterative and exploratory, as is customary for a narrative review, and no review protocol was registered. Records were not prospectively deduplicated or entered into a PRISMA-style screening database. Consequently, reliable retrospective counts of records identified, deduplicated, and screened cannot be provided. A PRISMA flow diagram was not constructed because reconstructing these counts after completion of the review could imply a degree of systematic precision that the search process did not have. Sixty-one sources were ultimately included in the narrative synthesis.

Methodological quality and clinical relevance were assessed qualitatively. The assessment considered study design, risk of bias, directness, consistency, precision, population applicability, recency, and the certainty assigned by guideline panels. Systematic reviews and current graded guidelines were prioritized over narrative reviews and older expert opinion.

Recommendations supported mainly by observational studies, indirect evidence, or expert consensus are identified as uncertain. Surveillance estimates were interpreted in relation to their sampling frames, isolate-submission practices, laboratory breakpoints, geographic coverage, travel exposure, and study period. Such estimates were used to inform, rather than independently determine, empirical treatment recommendations.

## 3. General Principles of Antibiotic Therapy in Gastrointestinal Infections

### 3.1. When Not to Use Antibiotics

Viral infections are the most common cause of acute diarrhea, particularly in high-income countries, and include pathogens such as norovirus, rotavirus, and adenovirus. In the United States, norovirus is the leading cause of gastroenteritis and is especially prevalent in closed settings such as cruise ships, nursing homes, dormitories, and hospitals. A decline in adult rotavirus-associated gastroenteritis has been observed following the introduction of pediatric rotavirus vaccination. In these cases, management is primarily supportive, based on oral or intravenous rehydration, with no benefit from antibiotic therapy. In patients with moderate to severe diarrhea, the primary therapeutic goal is the correction and maintenance of fluid and electrolyte balance, which may be life-saving in elderly individuals, patients with comorbidities, and neonates [5,9].

Antibiotics are not recommended for mild, tolerable diarrheal symptoms that do not interfere with daily activities. In most individuals with acute watery diarrhea and no recent international travel, empirical antimicrobial therapy is not indicated and should also be avoided in cases of persistent watery diarrhea lasting more than 14 days. Self-limiting watery diarrhea is typically characterized by mild to moderate symptoms, absence of high fever, and spontaneous resolution within a few days. In such cases, antibiotics do not significantly shorten disease duration and may instead disrupt the intestinal microbiota, promoting complications such as antimicrobial resistance and opportunistic infections [5,6,9].

### 3.2. When to Consider Antibiotics

Antibiotic therapy should be considered only when the expected clinical benefit outweighs the risks of adverse effects, disruption of the intestinal microbiota, and selection of antimicrobial resistance. The decision should integrate disease severity, the presence of systemic toxicity or sepsis, stool characteristics, duration of illness, patient age and immune status, recent travel or outbreak exposure, and the likelihood of a treatable bacterial pathogen. Whenever feasible, diagnostic specimens should be obtained before treatment so that empirical therapy can be narrowed, changed, or discontinued once an etiology is established [5,6].

Empirical therapy is therefore reserved for selected high-risk presentations rather than used routinely in acute diarrhea. In immunocompetent children and adults with bloody diarrhea, antimicrobial treatment is generally not recommended while diagnostic results are pending, except in the following situations:

- Infants younger than 3 months of age in whom a bacterial etiology is suspected;
- Ill immunocompetent patients with fever, abdominal pain, bloody diarrhea, and clinical features of bacillary dysentery suggestive of Shigella infection;
- Patients with recent international travel who present with a body temperature ≥ 38.5 °C and/or signs of sepsis [5].

Empirical antibacterial treatment should also be considered in immunocompromised patients with severe illness and bloody diarrhea [5]. When empirical therapy is justified in adults, either azithromycin or a fluoroquinolone may be selected according to travel history, the suspected pathogen, contraindications, and local or travel-associated susceptibility patterns. Appropriate stool and, when indicated, blood cultures should be collected before antibiotic administration because treatment may reduce culture yield [5,6]. In children, fluoroquinolones are not routinely recommended, but may be used for selected severe or resistant infections when the expected benefit outweighs potential toxicity and suitable alternatives are unavailable; age-specific guidance and susceptibility results should be followed.

Once a clinically plausible pathogen is identified, therapy should become pathogen-directed. Antibiotics are beneficial in only a minority of acute diarrheal illnesses, but may be important for shigellosis, selected cases of salmonellosis or campylobacteriosis, cholera, enteric fever, and CDI; they may be lifesaving in bacteremic salmonellosis and other severe invasive infections [9]. Adjunctive loperamide may be considered in adequately hydrated, immunocompetent adults with moderate-to-severe non-dysenteric travelers’ diarrhea, but it should not be used as monotherapy in patients with fever or bloody diarrhea.

### 3.3. Factors Influencing Therapeutic Choice

Empirical selection should use the most local and recent susceptibility information available, while recognizing that surveillance data describe tested isolates rather than the probability that an individual patient harbors a resistant organism.

Systems such as the US National Antimicrobial Resistance Monitoring System integrate isolates from people, food, and food-producing animals and are valuable for detecting trends. However, reported resistance proportions are sensitive to who is cultured, referral and submission practices, laboratory breakpoints, travel-related case mix, and the period and region sampled. These limitations preclude direct extrapolation of a single regional percentage to every clinical setting. The consistent signal is more important than any isolated estimate: fluoroquinolone resistance is high in *Campylobacter* and increasingly relevant in *Salmonella* and *Shigella*, while azithromycin susceptibility also varies. Empirical therapy should therefore reflect the patient’s exposure geography and local antibiogram, and culture with susceptibility testing should be obtained when treatment failure, invasive disease, or public-health implications are plausible [10,11,12].

The decision to initiate empirical antibiotic therapy in infectious diarrhea should be guided by disease severity and the availability of diagnostic testing. Collection of appropriate diagnostic specimens should be prioritized, as antibiotic administration may affect culture yield.

A particular management challenge is posed by STEC, in which the risks of empirical antibiotic therapy may outweigh potential benefits [6].

The management of travelers’ diarrhea requires special consideration because resistance patterns vary by destination and surveillance findings may not be directly transferable to an individual patient [6]. A multinational surveillance study examined enteropathogens isolated from 859 travelers returning from 103 destinations. Among *Campylobacter* isolates, fluoroquinolone resistance was reported in 88% of isolates associated with travel to South-Central Asia and 80% of those associated with travel to Southeast Asia. Macrolide resistance was lower but remained clinically relevant. Among Shigella isolates from travelers returning from South America, macrolide non-susceptibility was reported in 78% [12]. These estimates should modify rather than determine empirical therapy because the sampled travelers and submitted isolates may not represent all infections acquired in those regions. In practice, a consistent regional resistance signal should reduce reliance on an agent likely to be inactive, while disease severity, the suspected pathogen, patient-specific contraindications, local guidance, and the availability of culture and susceptibility testing should determine the final treatment decision.

## 4. Pathogen-Targeted Antibiotic Therapy

### 4.1. Salmonellosis

Salmonellosis is caused by *Salmonella enterica* and encompasses both non-typhoidal salmonellosis, which typically presents as acute gastroenteritis, and enteric fever caused by the typhoidal serovars *Salmonella Typhi* and *Salmonella Paratyphi*. Infection should be suspected following the consumption of raw or undercooked meat, poultry, or eggs; unpasteurized milk, dairy products, fruit juices, or other beverages; and contaminated vegetables, particularly leafy greens and sprouts. Additional epidemiological risk factors include exposure to foodborne outbreaks in restaurants, hotels, or cruise ships; contact with reptiles or young poultry; swimming in or drinking untreated freshwater; and travel to resource-limited settings [5].

The clinical presentation of non-typhoidal salmonellosis commonly includes acute diarrhea, which may be watery or bloody, abdominal cramps, fever, nausea, and occasionally vomiting. Although the infection is usually self-limiting, severe dehydration, bacteremia, sepsis, and extraintestinal complications may occur, particularly in infants, older adults, immunocompromised individuals, and patients with relevant comorbidities [5,6]. Enteric fever generally presents as a systemic febrile illness and may be accompanied by abdominal symptoms, diarrhea, constipation, hepatosplenomegaly, or neurological manifestations.

When microbiological confirmation is required, stool culture remains the standard diagnostic method for acute non-typhoidal salmonellosis. If a culture-independent molecular assay is positive, reflex stool culture should be considered to obtain an isolate for serotyping and antimicrobial susceptibility testing. Blood cultures are indicated in patients with suspected bacteremia, sepsis, enteric fever, or extraintestinal infection. For *Salmonella Typhi* and *Salmonella Paratyphi*, appropriate specimens may include blood, stool, bone marrow, and, in selected circumstances, duodenal fluid [5]. Antimicrobial susceptibility testing is particularly important when treatment is indicated because resistance patterns vary geographically and may be influenced by previous antimicrobial exposure and travel history.

Routine follow-up stool testing is generally unnecessary after clinical resolution. However, clearance testing may be required by local public health authorities, particularly for food handlers, healthcare workers, childcare personnel, or other individuals employed in settings associated with a high risk of transmission [5].

The cornerstone of treatment for uncomplicated non-typhoidal salmonellosis is supportive care, including oral or intravenous rehydration and correction of electrolyte abnormalities. Antibiotic therapy is not recommended for otherwise healthy patients with mild or moderate, self-limiting gastroenteritis because it generally does not shorten the duration of illness and may prolong fecal carriage, disrupt the intestinal microbiota, and promote antimicrobial resistance [9,13].

Antibiotic treatment should be reserved for severe disease, systemic toxicity, bacteremia, sepsis, extraintestinal infection, or increased risk of invasion. Evidence on duration is limited and is derived largely from observational cohorts and extrapolation from other bloodstream infections rather than trials specific to non-typhoidal *Salmonella*. In a clinically stable patient with uncomplicated bacteremia, rapid response, clearance of blood cultures, and no endovascular or focal infection, some contemporary practice guidance supports 5–7 days of active therapy. Longer courses are appropriate for immunocompromised patients, persistent bacteremia, endovascular infection, meningitis, osteoarticular infection, or other deep foci. Agent and duration should be guided by susceptibility, source control, host factors, and clinical response [5,9,13].

Unlike uncomplicated non-typhoidal gastroenteritis, enteric fever caused by *Salmonella Typhi* or *Salmonella Paratyphi* always requires antimicrobial treatment. Regimens and durations are summarized in Table 1, but local susceptibility data and the site of infection take precedence over a fixed schedule [4,9,14].

Evidence basis: Recommendations for enteric fever and invasive *Salmonella* infection are derived from clinical guidelines, comparative studies, and observational cohorts. Evidence for the exact duration of therapy in uncomplicated non-typhoidal *Salmonella* bacteremia is limited and is based mainly on observational evidence and extrapolation from other bloodstream infections.

Patients with persistent carriage of *Salmonella Typhi* should receive prolonged, susceptibility-guided antimicrobial therapy. Because chronic carriage is frequently associated with gallbladder colonization and cholelithiasis, evaluation for biliary disease is warranted when eradication therapy fails, and cholecystectomy may be considered in selected cases [9].

In pediatric patients, antimicrobial therapy is recommended for infants younger than 3 months because of their increased risk of bacteremia and other invasive complications. Third-generation cephalosporins, particularly ceftriaxone, are generally preferred for severe or invasive infections, whereas azithromycin may represent an effective oral alternative in selected patients, depending on clinical severity and local antimicrobial susceptibility patterns [9,13,14].

### 4.2. Shigellosis

Shigellosis is an acute enteric infection caused by *Shigella* spp., a group of highly contagious pathogens transmitted primarily by the fecal–oral route. Because ingestion of only a small number of organisms may cause disease, person-to-person transmission occurs readily, particularly in settings involving close contact or inadequate sanitation. Risk factors include travel to resource-limited regions, consumption of contaminated food or untreated water, exposure during foodborne or waterborne outbreaks, attendance or employment in childcare facilities, institutional residence, homelessness, and sexual practices involving oral–anal contact. Young children are particularly susceptible to infection and frequently contribute to secondary household and community transmission [5,9].

The clinical manifestations range from mild, self-limiting watery diarrhea to severe inflammatory diarrhea or dysentery. Typical symptoms include fever, abdominal cramps, tenesmus, and frequent small-volume stools containing blood or mucus. Nausea, vomiting, and dehydration may also occur. Although most infections resolve without complications, severe disease, bacteremia, toxic megacolon, neurological manifestations, and other extraintestinal complications may develop, particularly in young children, older adults, malnourished patients, and immunocompromised individuals. Infection with *Shigella dysenteriae* type 1 is of particular concern because this organism produces Shiga toxin and may be associated with HUS [5,6].

Microbiological confirmation is recommended in patients with bloody diarrhea, severe or prolonged illness, suspected outbreaks, or risk factors for antimicrobial-resistant infection. Diagnosis can be established using stool culture or a nucleic acid amplification test. When a culture-independent assay detects *Shigella*, reflex stool culture should be performed whenever possible to obtain an isolate for species identification, public health surveillance, and antimicrobial susceptibility testing. Culture and susceptibility testing are particularly important when antibiotic therapy is being considered because multidrug-resistant strains are increasingly prevalent [5].

The cornerstone of management is adequate oral or intravenous rehydration and correction of electrolyte abnormalities. Antimotility agents, including loperamide and diphenoxylate–atropine, should be avoided in patients with dysentery because they may worsen the clinical course or delay pathogen clearance. Antibiotic therapy is not required for every patient, as mild shigellosis is frequently self-limiting. Nevertheless, appropriate treatment can shorten the duration of fever and diarrhea, reduce fecal shedding, and limit person-to-person transmission [9,15,16].

Antibiotic therapy should therefore be considered for patients with dysentery, severe or prolonged disease, systemic toxicity, sepsis, or extraintestinal infection. Treatment is also appropriate for individuals at increased risk of complications, including young children, older or frail adults, malnourished patients, and immunocompromised individuals. From a public health perspective, antibiotics may additionally be indicated during outbreaks or when reducing transmission is particularly important, such as among food handlers and people living or working in childcare, healthcare, or long-term care facilities. Whenever possible, antimicrobial selection should be guided by culture and susceptibility results, local resistance patterns, previous antibiotic exposure, and travel history [9,15,16].

The global emergence of multidrug-resistant and extensively drug-resistant (XDR) *Shigella* has substantially complicated empirical treatment. The US Centers for Disease Control and Prevention defines XDR *Shigella* as resistant to all commonly recommended empirical and alternative agents, namely azithromycin, ciprofloxacin, ceftriaxone, trimethoprim–sulfamethoxazole, and ampicillin. Populations disproportionately affected by resistant infections include men who have sex with men, people experiencing homelessness, individuals living with HIV, and recent international travelers [6].

In patients with risk factors for resistant infection, stool culture and antimicrobial susceptibility testing should be obtained before treatment whenever clinically feasible. Regional surveillance findings, including high macrolide non-susceptibility among some travel-associated isolates, should modify but not independently determine empirical therapy. For example, a high regional resistance signal should prompt clinicians to obtain a viable isolate, review current destination-specific and local guidance, and avoid assuming that azithromycin will be active. The initial regimen should also reflect illness severity, host factors, and the availability of alternative agents. Treatment should be modified as soon as susceptibility results become available. Because evidence supporting the optimal treatment of extensively drug-resistant Shigella remains limited, severe, invasive, or treatment-refractory infections should be managed in consultation with infectious-disease and public-health specialists. Intravenous carbapenems may be considered for hospitalized patients with severe, extensively drug-resistant infections when supported by the anticipated or confirmed susceptibility profile, but routine empirical use is not recommended [5,6,12]. Recommended antimicrobial regimens and treatment considerations for shigellosis are summarized in Table 2 [4,9,14,15,17].

Evidence basis: The clinical benefit of antibiotic treatment for susceptible shigellosis is supported by clinical trials and guideline recommendations. Selection of treatment for multidrug-resistant or extensively drug-resistant *Shigella* relies mainly on surveillance data, case series, and expert consensus.

### 4.3. Campylobacteriosis

Campylobacteriosis is an acute enteric infection caused predominantly by *Campylobacter jejuni*, although other species, including *C*. *coli*, *C*. *fetus*, *C*. *upsaliensis*, and *C*. *lari*, may also cause human disease. It is among the most common causes of bacterial gastroenteritis worldwide. Transmission occurs primarily through the consumption of raw or undercooked poultry or foods cross-contaminated during preparation. Other risk factors include the consumption of unpasteurized milk or dairy products, exposure to untreated or contaminated water, international travel, contact with infected dogs or cats, and occupational or recreational exposure to poultry, livestock, or animals at petting zoos. Although outbreaks have been associated with contaminated food, raw milk, and water, most infections are sporadic, and poultry remains the most frequently implicated source [5,18].

Following an incubation period of approximately 2–5 days, campylobacteriosis typically presents with acute diarrhea, which may be watery or bloody, accompanied by fever, abdominal cramps, nausea, malaise, and occasionally vomiting. Abdominal pain may be sufficiently severe to mimic acute appendicitis or inflammatory bowel disease. Most infections are self-limiting and resolve within approximately one week; however, severe colitis, dehydration, bacteremia, sepsis, and extraintestinal infection may occur, particularly in vulnerable patients [5,6,19].

Microbiological testing should be considered in patients with bloody diarrhea, severe or prolonged symptoms, systemic manifestations, suspected outbreaks, or an increased risk of complications. Diagnosis can be established through stool culture or a culture-independent diagnostic test, including nucleic acid amplification testing. Although molecular assays provide rapid and sensitive detection, culture confirmation of positive specimens remains important for antimicrobial susceptibility testing, epidemiological surveillance, and molecular subtyping. Susceptibility testing is especially relevant when treatment is indicated because fluoroquinolone resistance is increasingly common and resistance patterns vary geographically [5,19].

The cornerstone of management is supportive care, including adequate oral or intravenous rehydration and correction of electrolyte abnormalities. Routine antibiotic therapy is not recommended for mild or uncomplicated infection because most patients recover spontaneously and the clinical benefit of treatment is generally modest. When indicated, antimicrobial therapy is most effective when initiated early in the course of illness [4,9,19].

Antibiotic treatment should be considered for patients with severe disease, including high fever, dysentery, severe abdominal pain, worsening or persistent symptoms, substantial volume depletion, bacteremia, sepsis, or other evidence of extraintestinal infection. Treatment may also be appropriate for individuals at increased risk of severe or invasive disease, particularly adults aged 65 years or older, pregnant women, immunocompromised patients, and individuals with significant underlying comorbidities. In these populations, the decision to initiate therapy should be based on the severity and duration of symptoms, host-related risk factors, and the likelihood of antimicrobial resistance [4,9,19].

Macrolides, particularly azithromycin, are generally preferred for severe enteritis because of their efficacy and the high prevalence of fluoroquinolone resistance (Table 3). Fluoroquinolones should be used only when susceptibility is demonstrated or reliable local surveillance indicates low resistance. Invasive or extraintestinal infection requires species identification, site-specific assessment, blood-culture susceptibility testing, and infectious-disease consultation. Aminoglycosides or carbapenems are not general alternatives for enteritis; they may be selected only for exceptional systemic infection caused by a susceptible isolate when the infection site, host factors, and lack of safer active options justify their use [5,6,19].

Evidence basis: Macrolide treatment of clinically significant *Campylobacter enteritis* is supported by clinical trials and guideline recommendations. Regimens for invasive or extraintestinal infection rely predominantly on observational evidence and expert consultation.

Although uncommon, campylobacteriosis may be followed by clinically important post-infectious complications. These include reactive arthritis, post-infectious irritable bowel syndrome, and Guillain–Barré syndrome. Guillain–Barré syndrome usually develops several days to weeks after the gastrointestinal infection and is attributed to molecular mimicry between bacterial lipooligosaccharides and gangliosides expressed on peripheral nerves. Febrile seizures may also occur, particularly in young children with high fever [6,9,19].

### 4.4. Escherichia coli

*Escherichia coli* comprises a heterogeneous group of Gram-negative bacteria, most of which are harmless components of the intestinal microbiota. However, several diarrheagenic pathotypes possess distinct virulence factors and cause gastrointestinal disease. The principal pathotypes include enterotoxigenic *E. coli* (ETEC), STEC, including enterohemorrhagic *E. coli* (EHEC), enteropathogenic *E. coli* (EPEC), enteroaggregative *E. coli* (EAEC), enteroinvasive *E. coli* (EIEC), and diffusely adherent *E. coli* (DAEC). Their clinical manifestations range from self-limiting watery diarrhea to invasive hemorrhagic colitis and life-threatening systemic complications.

ETEC is a leading bacterial cause of travelers’ diarrhea and typically produces acute watery diarrhea accompanied by abdominal cramps, nausea, and occasionally low-grade fever. EPEC and EAEC are also associated with travelers’ diarrhea and may cause persistent symptoms, particularly in children and immunocompromised patients. EIEC produces an invasive inflammatory illness that may resemble shigellosis. By contrast, STEC infection commonly presents with severe abdominal cramps and bloody diarrhea, often with little or no fever, and may progress to HUS through the systemic effects of Shiga toxin.

The diagnosis of diarrheagenic *E. coli* infection is increasingly based on multiplex nucleic acid amplification tests. When STEC is suspected, stool specimens should be tested for Shiga toxin or the genes encoding it and cultured for E. coli O157. Shiga toxin-positive specimens and presumptive O157 isolates should be forwarded to an appropriate public health laboratory for further characterization. Rapid identification of STEC is particularly important because the administration of antibiotics or antimotility agents may adversely affect clinical outcomes [7,20,21,22,23,24].

Management of non-STEC diarrheagenic *E. coli* infection is primarily guided by clinical severity. In travelers’ diarrhea, severity should be classified according to its functional impact rather than stool frequency alone. Mild illness is tolerable and does not interfere with planned activities; moderate illness is distressing or interferes with activities; and severe illness is incapacitating or prevents normal activities. All dysentery should be considered severe. Persistent travelers’ diarrhea is not a separate severity category but is defined by a duration of at least 14 days and requires additional diagnostic evaluation, particularly for parasitic causes [1].

Antibiotics are not recommended for mild travelers’ diarrhea. For moderate illness, antibiotic therapy may be considered according to the patient’s symptoms, comorbidities, and preferences, whereas treatment is generally advised for severe disease. Azithromycin is preferred for severe, febrile, or dysenteric diarrhea and in regions where fluoroquinolone resistance is prevalent. Rifaximin may be considered for moderate non-invasive diarrhea but should not be used when invasive disease is suspected, including in patients with fever or bloody stools. Fluoroquinolones represent an alternative only when local susceptibility patterns support their use, with additional caution warranted because of their adverse-effect profile and increasing global resistance [1,7,20,21,22].

Single-dose antibiotic regimens are effective for many patients with travelers’ diarrhea and may improve adherence. If symptoms have not resolved within 24 h after a single dose, treatment may be continued using the corresponding multidose regimen for up to three days. In adults, loperamide may be used alone for mild or moderate non-invasive diarrhea or as an adjunct to antibiotics. However, antimotility agents should not be used as monotherapy in patients with fever or bloody diarrhea and must be avoided when STEC infection is suspected or confirmed [1,7,20,21,22].

In contrast to ETEC and other non-STEC pathotypes, antibiotic therapy should be avoided in patients with suspected or confirmed STEC infection, particularly infections caused by *E. coli* O157, strains producing Shiga toxin 2, or strains for which the toxin genotype is unknown. Antimicrobial exposure may increase Shiga toxin production or release and has been associated with an increased risk of HUS. Antimotility agents should likewise be avoided because delayed intestinal transit may increase toxin exposure and the risk of complications, including HUS, toxic megacolon, and neurological involvement [7,23,24].

The management of STEC infection is therefore supportive and should emphasize prompt assessment of hydration status, appropriate fluid and electrolyte replacement, and close monitoring for the development of HUS. Early intravenous fluid administration may reduce the risk of renal complications in children with STEC infection. Patients should be monitored for anemia, thrombocytopenia, acute kidney injury, and neurological manifestations, particularly during the first two weeks after diarrhea onset [7,23,24].

Resistance among ETEC and other diarrheagenic *E. coli* limits empirical treatment options for travelers’ diarrhea. Fluoroquinolone resistance is particularly relevant after travel in South and Southeast Asia and is increasingly reported elsewhere. Trimethoprim-sulfamethoxazole and ampicillin are also unreliable empirical choices in many settings. These surveillance findings should modify rather than dictate treatment. In a patient with severe, febrile, or dysenteric diarrhea acquired in a region with consistently high fluoroquinolone resistance, azithromycin is generally the preferred empirical option, provided that STEC has been considered and local or destination-specific guidance does not indicate substantial macrolide resistance. Fluoroquinolones should be reserved for settings in which susceptibility is documented or reliable contemporary data indicate that activity is likely. Rifaximin and rifamycin SV are options only for non-invasive disease because they do not provide adequate systemic treatment for *Campylobacter*, *Salmonella*, *Shigella*, or invasive *E. coli*. Culture and susceptibility testing should be pursued when illness is severe, invasive disease is suspected, initial treatment fails, or the result has public-health implications [4,7,11,12,20,21,22]. Recommended strategies are summarized in Table 4 [4,7,11,12,20,21,22].

Evidence basis: Antibiotic treatment of moderate-to-severe travelers’ diarrhea is supported by randomized trials and guideline recommendations. The recommendation to avoid antibiotics in STEC infection is based mainly on observational studies and pooled analyses because randomized exposure trials would be ethically problematic.

### 4.5. Vibrio Cholerae

Cholera is an acute secretory diarrheal disease caused by toxigenic strains of *Vibrio cholerae*, predominantly serogroups O1 and O139. Transmission occurs through the ingestion of contaminated food or water and is strongly associated with inadequate access to safe water, sanitation, and hygiene. Although many infections are asymptomatic or mild, severe cholera is characterized by the abrupt onset of profuse, painless watery diarrhea—classically described as “rice-water” stool—often accompanied by vomiting and leg cramps. Rapid fluid losses can result in severe dehydration, electrolyte disturbances, metabolic acidosis, hypovolemic shock, acute kidney injury, and death within hours if treatment is delayed [9,25].

Immediate fluid and electrolyte replacement is the cornerstone of management and has the greatest effect on survival. Oral rehydration solution should be initiated as soon as cholera is suspected and continued to replace ongoing losses. Patients with severe dehydration, shock, altered consciousness, uncontrollable vomiting, or an inability to drink require rapid intravenous rehydration, preferably with Ringer’s lactate, followed by oral rehydration as soon as tolerated. Breastfeeding and age-appropriate feeding should be continued, and zinc supplementation should be provided to children where recommended. Antibiotics must always be regarded as an adjunct to, and never a substitute for, prompt and adequate rehydration [9,25].

Most patients with mild cholera recover with rehydration alone and do not require antimicrobial treatment, including during outbreaks. Antibiotic therapy is recommended primarily for patients with severe cholera or severe dehydration and may also be considered for hospitalized patients with moderate dehydration who continue to pass large volumes of watery stool during rehydration. In appropriately selected patients, effective antimicrobial treatment reduces the duration of diarrhea, stool volume, fluid requirements, bacterial shedding, and length of hospitalization [7,9,25,26].

The selection of an antimicrobial agent should be based on local or outbreak-specific susceptibility data because resistance patterns may change rapidly. A single oral dose of doxycycline is generally effective and convenient for susceptible infections. Azithromycin is an effective alternative and is often preferred for pregnant women, children, and patients infected in regions where resistance to tetracyclines or fluoroquinolones is prevalent. Other options, including tetracycline, ciprofloxacin, or selected cephalosporins, may be considered according to antimicrobial susceptibility, patient characteristics, contraindications, and local availability. Single-dose regimens are preferred when supported by susceptibility data, although multidose treatment for up to three days may be required with certain agents or in cases of an inadequate clinical response [7,27,28,29,30].

Empirical antibiotic therapy is not recommended for most patients presenting with undifferentiated acute watery diarrhea. However, treatment may be initiated after rehydration in patients with severe illness and a strong epidemiological suspicion of cholera, particularly during a confirmed outbreak or following travel to an affected area, without awaiting microbiological confirmation. The empirical agent should reflect current local susceptibility data and should be modified if subsequent testing identifies resistance. Antimotility agents are not recommended in severe cholera because they do not address the potentially life-threatening fluid losses and may interfere with appropriate clinical monitoring [5,22].

Microbiological testing is particularly important when cholera is suspected outside a recognized outbreak, in the first cases of a possible outbreak, in patients with severe disease, and for antimicrobial-resistance surveillance. Confirmation is based on the isolation and identification of toxigenic *V. cholerae* O1 or O139 from a stool specimen or rectal swab. Culture on selective media, including thiosulfate–citrate–bile salts–sucrose agar, remains a standard diagnostic method. Polymerase chain reaction and other molecular techniques can provide rapid and sensitive detection, whereas rapid diagnostic tests may support outbreak investigation but generally require confirmation according to public health protocols [5,22].

Routine antibiotic prophylaxis for household or community contacts is not recommended. Although chemoprophylaxis may transiently reduce secondary infections, it does not provide sustained protection and may promote antimicrobial resistance, cause adverse effects, and divert resources from more effective interventions. Prevention should instead focus on rapid case identification, access to safe water, adequate sanitation, hand hygiene, food safety, health education, and vaccination when indicated [31]. Recommended antimicrobial regimens and treatment considerations for cholera are summarized in Table 5 [7,9,25,26,27,28,29,30,31].

Evidence basis: The effect of antibiotics in shortening clinically significant cholera is supported by randomized trials and systematic review evidence. Selection of a specific agent increasingly depends on surveillance data, local susceptibility patterns, and guideline consensus.

### 4.6. Less Common Bacterial Causes of Acute Diarrhea

*Yersinia enterocolitica* usually causes self-limited enterocolitis and does not require antibiotics. Treatment should be reserved for severe or systemic disease, bacteremia, or high-risk hosts, and should be guided by susceptibility testing. Trimethoprim-sulfamethoxazole is commonly active; third-generation cephalosporins or fluoroquinolones may be considered for invasive infection, while severe sepsis requires specialist management [5,6].

*Aeromonas* spp. and *Plesiomonas shigelloides* usually cause self-limited watery diarrhea. Antibiotics are generally unnecessary but may be considered for severe, persistent, dysenteric, or extraintestinal disease and for immunocompromised patients. Because species identification and susceptibility vary, treatment should be individualized; azithromycin, ciprofloxacin, or trimethoprim-sulfamethoxazole may be options when an active agent is required [5,6].

Non-cholera *Vibrio* infections range from self-limited gastroenteritis to wound infection and fulminant septicemia, particularly in patients with chronic liver disease or iron overload. Mild gastroenteritis is treated supportively. Severe *V. vulnificus* or *V. parahaemolyticus* infection requires urgent culture, susceptibility-guided systemic therapy, assessment for wound source control, and infectious-disease consultation; regimens depend on species, syndrome, and local guidance [5,6].

### 4.7. Clostridioides Difficile Infection

CDI is a major cause of healthcare-associated diarrhea and one of the most clinically important complications of antimicrobial exposure. Antibiotics disrupt colonization resistance within the intestinal microbiota, allowing toxigenic *C. difficile* strains to proliferate and produce toxins A and B. Additional risk factors include advanced age, recent hospitalization or residence in a long-term care facility, immunosuppression, serious underlying disease, and a previous episode of CDI.

CDI is defined by compatible clinical manifestations—typically three or more new, unformed stools within 24 h—together with laboratory evidence of toxigenic *C. difficile* or free toxin in the stool, in the absence of another likely explanation. Testing should be restricted to symptomatic patients and performed only on unformed stool, as nucleic acid amplification tests may detect asymptomatic colonization and lead to overdiagnosis. Depending on institutional diagnostic-stewardship practices, multistep algorithms combining glutamate dehydrogenase, toxin enzyme immunoassay, and nucleic acid amplification testing may improve diagnostic specificity. CDI may also be diagnosed by the demonstration of pseudomembranous colitis during endoscopy, surgery, or histopathological examination. Repeat testing to document cure is not recommended because patients may remain colonized after clinical recovery [32].

Initial management should include assessment of disease severity, correction of fluid and electrolyte abnormalities, discontinuation of unnecessary antimicrobials, and review of other medications that may contribute to diarrhea or impair recovery. Treatment selection should consider disease severity, previous CDI episodes, recurrence risk, drug availability, and patient-specific factors.

For an initial non-fulminant CDI episode, the 2021 IDSA/SHEA focused update suggests fidaxomicin rather than a standard course of oral vancomycin, while recognizing that implementation depends on available resources. Oral vancomycin remains an acceptable evidence-based alternative. Fidaxomicin and vancomycin achieve similar initial clinical response rates, but fidaxomicin is associated with fewer recurrences. Its incremental benefit may therefore be particularly relevant in patients at increased risk of recurrence, including older adults, immunocompromised patients, and those with previous CDI. Cost, reimbursement, formulary restrictions, and local availability may nevertheless limit its use, particularly in low- and middle-income settings. Where fidaxomicin is unavailable or financially inaccessible, oral vancomycin should be used in accordance with national or local guidance rather than delaying effective treatment. Decisions should reflect both the individual risk of recurrence and the resources of the healthcare system [33,34,35].

Metronidazole is not a preferred first-line treatment for an initial CDI episode, including non-severe disease. It should be reserved for the exceptional situation in which fidaxomicin and oral vancomycin are unavailable, and repeated or prolonged courses should be avoided because of lower efficacy and cumulative neurotoxicity. This hierarchy should not be interpreted as therapeutic equivalence among the three agents [33,35,36].

#### 4.7.1. Severe and Fulminant Clostridioides Difficile Infection

Severe CDI is defined by leukocytosis or increased serum creatinine without hypotension, shock, ileus, or toxic megacolon. It is treated with the same standard-dose fidaxomicin or oral vancomycin regimens used for other non-fulminant initial episodes. Fulminant CDI is defined by hypotension or shock, ileus, toxic megacolon, or other evidence of critical illness and requires urgent medical and surgical assessment.

For fulminant CDI, high-dose oral or nasogastric vancomycin 500 mg four times daily is recommended, with intravenous metronidazole 500 mg every 8 h, particularly when ileus may limit intraluminal delivery. Rectal vancomycin should be considered when ileus is present. These intensified regimens should not be used solely because laboratory criteria classify an episode as severe [34,35].

Fidaxomicin is not standard therapy for fulminant CDI because patients with fulminant disease were generally excluded from pivotal trials. Intravenous tigecycline is also not a routine guideline-recommended regimen. It may be considered only as salvage therapy in selected refractory cases after infectious-disease and surgical consultation; supporting evidence is limited to small, uncontrolled observational series, and its use must not delay surgery or other source-control measures [35,37].

#### 4.7.2. Recurrent Clostridioides Difficile Infection

Recurrent CDI is generally defined as the reappearance of compatible symptoms and a positive diagnostic test within 2–8 weeks after resolution of a previous episode. Approximately 20–25% of patients experience recurrence after an initial episode, and the probability of further recurrence increases substantially with each subsequent episode. Important risk factors include older age, immunosuppression, severe CDI, continued exposure to non-CDI antibiotics, and previous recurrence [32].

For a first recurrence, standard-course or extended-pulsed fidaxomicin is generally preferred because it reduces the likelihood of an additional recurrence. A tapered and pulsed oral vancomycin regimen is an effective alternative, particularly when fidaxomicin is unavailable or when vancomycin was not used for the initial episode. By introducing antibiotic-free intervals, the tapered-pulsed strategy allows residual spores to germinate and exposes the resulting vegetative organisms to subsequent vancomycin doses [32,38].

Patients with multiple recurrences require an individualized strategy. Options include fidaxomicin, a tapered and pulsed vancomycin regimen, or oral vancomycin followed by rifaximin. The choice should account for previous treatment responses, recurrence risk, drug accessibility, and the need to restore microbiome-mediated colonization resistance [32,38,39].

Bezlotoxumab, a human monoclonal antibody directed against toxin B, has been shown to reduce recurrence when administered as a single intravenous infusion alongside standard antimicrobial therapy, particularly in patients aged 65 years or older, immunocompromised individuals, and those with severe CDI or a recent recurrence. It does not treat the active infection and should only be used as an adjunct to appropriate antimicrobial therapy. Its clinical use is dependent on regional availability, and caution is required in patients with congestive heart failure [32,39].

Microbiota-restoration therapy is an important option for preventing further episodes in patients with recurrent CDI after completion of appropriate antimicrobial treatment. Conventional fecal microbiota transplantation (FMT) is highly effective for appropriately selected patients with multiple recurrences, but it must be performed using rigorously screened donor material because of the potential transmission of infectious organisms and antimicrobial-resistance genes. Standardized microbiota-based products, administered rectally or orally, provide additional options in jurisdictions where they have received regulatory approval. These interventions are intended to prevent recurrence by restoring microbial diversity and colonization resistance; they are not substitutes for antimicrobial treatment of an active severe or fulminant episode [32,39].

Current guidelines do not support the routine use of probiotics for the primary prevention of CDI or the prevention of recurrence because efficacy remains uncertain and results vary substantially according to the organism, formulation, dose, and patient population. Although selected preparations containing *Saccharomyces boulardii* or *Lactobacillus* species have shown possible benefit in individual studies, probiotics may cause invasive infection in severely immunocompromised or critically ill patients and should not be routinely recommended [32].

#### 4.7.3. Surgical Management

Early surgical consultation is essential in patients with fulminant CDI accompanied by toxic megacolon, colonic perforation, peritonitis, an acute surgical abdomen, worsening organ failure, rising lactate levels, or progressive clinical deterioration despite optimal medical therapy. Delaying consultation until refractory shock or multiorgan failure develops may substantially worsen the prognosis.

Subtotal or total abdominal colectomy with preservation of the rectum and formation of an end ileostomy remains the standard operative approach for fulminant colitis requiring surgery. Diverting loop ileostomy with intraoperative colonic lavage and postoperative intracolonic vancomycin represents a colon-preserving alternative in carefully selected patients, although evidence supporting its comparative effectiveness remains limited. The choice of procedure should be individualized according to disease extent, physiological status, surgical expertise, and the likelihood of colonic recovery [40].

#### 4.7.4. Clinical Bottom Line

CDI management requires diagnostic stewardship, prevention of unnecessary non-CDI antibiotic exposure, and recurrence-risk assessment. Fidaxomicin is preferred for most initial and recurrent non-fulminant episodes when feasible, with oral vancomycin as an effective alternative. Metronidazole is reserved for non-severe disease only when both preferred agents are unavailable. Fulminant CDI requires high-dose oral or enteral vancomycin plus intravenous metronidazole, consideration of rectal vancomycin for ileus, and early surgical consultation. Tigecycline is a non-standard salvage option supported by very low-quality evidence. Table 6 summarizes this treatment hierarchy [32,33,35,36,38,39].

Evidence basis: Fidaxomicin and oral vancomycin for non-fulminant and recurrent CDI are supported by randomized trials and graded guideline recommendations. Recommendations for fulminant disease and salvage treatment rely more heavily on observational evidence and expert consensus. Microbiota-based interventions are supported by clinical trials and guidelines only for selected patients with recurrent CDI.

## 5. Antimicrobial Resistance

Antimicrobial resistance among enteric pathogens is an expanding threat to individual patient care, outbreak control, and global health security. Resistant gastrointestinal infections may result in treatment failure, prolonged symptoms and bacterial shedding, greater reliance on broad-spectrum or parenteral agents, increased hospitalization, and a higher risk of invasive complications. The epidemiology of resistance is highly dynamic and varies according to pathogen, antimicrobial class, geographical region, travel history, food-production practices, and local patterns of antimicrobial use [10,47].

Enteric bacteria acquire resistance through several mechanisms, including chromosomal mutations and horizontal transfer of mobile genetic elements such as plasmids, transposons, and integrons. These mechanisms may alter antimicrobial targets, reduce membrane permeability, increase drug efflux, or enable the production of antibiotic-inactivating enzymes. The intestinal microbiota provides an important reservoir for resistance genes, facilitating their exchange between commensal organisms and pathogenic bacteria. Inappropriate or unnecessary antimicrobial exposure intensifies the selective pressure favoring resistant strains and may promote their persistence and transmission [2,32,48,49].

Fluoroquinolone resistance is particularly important because these agents were historically used as first-line empirical therapy for travelers’ diarrhea and several invasive bacterial gastrointestinal infections. Resistance is now widespread among *Campylobacter jejuni* and has also increased among diarrheagenic *Escherichia coli*, *Shigella*, and *Salmonella*. Fluoroquinolone resistance among *Campylobacter* isolates is especially prevalent in South and Southeast Asia and is also reported in many other regions. Consequently, empirical fluoroquinolone treatment of returning travelers may be ineffective, particularly when the infection was acquired in a region with high resistance, while still exposing patients to adverse effects and further selective pressure [7,11,12,50].

Macrolides, particularly azithromycin, have become important alternatives for severe travelers’ diarrhea and infections caused by fluoroquinolone-resistant organisms. However, reduced macrolide susceptibility and clinically relevant resistance are increasingly reported in *Shigella* and *Campylobacter*. This trend is particularly concerning because azithromycin is one of the few convenient oral options available for children, pregnant women, and travelers returning from regions with extensive fluoroquinolone resistance [11,51].

Multidrug-resistant (MDR) and extensively drug-resistant phenotypes represent a further therapeutic challenge. MDR *Shigella* and non-typhoidal *Salmonella* may be resistant to several traditional oral agents, limiting empirical treatment options and increasing the potential need for parenteral therapy. Resistant infections may also be associated with delayed administration of effective treatment, prolonged bacterial shedding, transmission within communities or institutions, and a greater risk of invasive disease in vulnerable patients [10,15,35].

International travel contributes substantially to the acquisition and global dissemination of antimicrobial-resistant enteric bacteria. Travelers may become colonized with extended-spectrum β-lactamase-producing Enterobacterales after exposure to contaminated food, water, healthcare environments, or community reservoirs. Reported acquisition rates vary considerably according to destination and study population but may exceed 50% among travelers returning from high-prevalence regions. Antibiotic use during travel and the occurrence of travelers’ diarrhea further increase this risk, although colonization may also occur without antimicrobial exposure. Returning travelers can subsequently introduce resistant organisms and mobile resistance genes into households, communities, and healthcare settings [11,52,53,54].

The food-production system is another major component of this problem. Antimicrobial use in livestock and other food-producing animals creates selective pressure that can favor resistant zoonotic bacteria, including *Campylobacter* and non-typhoidal *Salmonella*. Resistant organisms or resistance genes may then reach humans through the food chain, direct animal contact, or environmental contamination. The interaction between human, animal, food, and environmental reservoirs highlights the need for a coordinated One Health response [10,45].

The increasing prevalence and geographical variability of resistance have shifted clinical practice away from indiscriminate empirical prescribing and toward selective, evidence-based treatment. Antibiotics should be reserved for infections in which a meaningful clinical or public health benefit is expected, particularly severe, invasive, persistent, or high-risk disease. Whenever possible, therapy should be guided by microbiological identification, antimicrobial-susceptibility results, travel history, and current local surveillance data [4,5,55].

Antimicrobial resistance in gastrointestinal infections is therefore a multifactorial problem that cannot be addressed through prescribing interventions alone. Effective control requires rational antimicrobial use in human and veterinary medicine, improved access to rapid and reliable diagnostics, strengthened laboratory capacity, coordinated international surveillance, safe water and sanitation, infection-prevention measures, vaccination, and regulation of antimicrobial use throughout the food-production chain [56,57,58].

## 6. Antimicrobial Stewardship

Antimicrobial stewardship is a coordinated approach to improving and measuring antimicrobial use in order to optimize clinical outcomes, minimize drug-related toxicity, preserve the intestinal microbiota, and limit the selection and transmission of resistant organisms. In gastrointestinal infections, stewardship is especially important because most episodes of acute diarrhea are viral or self-limiting and do not benefit from antibiotics. The objective is not simply to reduce prescribing but to ensure that patients who genuinely require treatment receive the most appropriate agent, dose, route, and duration without avoidable delay [8,49,52,58,59].

A fundamental component of stewardship is careful clinical assessment. Before prescribing antibiotics, clinicians should evaluate disease severity, hydration status, the presence of fever or dysentery, symptom duration, travel and exposure history, immune status, age, comorbidities, and evidence of sepsis or extraintestinal infection. Supportive care and close observation are appropriate for most otherwise healthy patients with mild, self-limiting illness. By contrast, prompt empirical treatment may be justified in selected patients with severe disease or a high risk of invasive infection, provided that appropriate diagnostic specimens are collected before treatment whenever feasible [5,59,60].

Diagnostic stewardship is closely linked to antimicrobial stewardship. Conventional methods, including stool culture and microscopic or antigen-based examination for parasites, remain important but may have limited sensitivity and often require 48–72 h or longer to provide definitive results. These delays may encourage empirical prescribing, particularly in severely ill or high-risk patients [4,5,61].

Multiplex molecular gastrointestinal panels can detect numerous bacterial, viral, and parasitic targets from a single stool specimen, often within a few hours. Their rapid turnaround time can support earlier pathogen-directed treatment, facilitate the discontinuation of unnecessary empirical therapy, identify infections in which antibiotics should be avoided, and improve infection-control decisions. These benefits depend on using the tests in clinically appropriate patients and ensuring that results are reviewed promptly [8,32].

Molecular results nevertheless require careful interpretation. Detection of microbial nucleic acid does not necessarily establish active disease because the identified organism may represent asymptomatic colonization, prolonged shedding, or nonviable material. Multiple organisms may be detected simultaneously, and the clinical significance of each finding may be uncertain. Results must therefore be interpreted in conjunction with the patient’s symptoms, epidemiological history, immune status, and pretest probability of infection [9,57].

Most multiplex panels identify pathogens but do not provide comprehensive phenotypic antimicrobial-susceptibility information. Although some assays detect selected resistance genes, the presence or absence of these markers does not always predict the complete susceptibility profile. Stool culture and antimicrobial-susceptibility testing remain necessary when treatment decisions, outbreak investigation, resistance surveillance, or public health reporting require a viable isolate. Molecular testing should therefore complement, rather than replace, conventional microbiology [4,55].

When empirical antibiotics are initiated, treatment should be reassessed as soon as clinical and microbiological information becomes available. Therapy should be discontinued if a bacterial indication is not confirmed, narrowed to the most targeted effective agent when a pathogen is identified, and administered for the shortest evidence-based duration. Additional stewardship measures include avoiding duplicate antimicrobial coverage, adjusting doses for age and renal or hepatic function, converting from intravenous to oral therapy when appropriate, and incorporating local antibiograms and travel-associated resistance data into empirical treatment protocols [4,49,52,59].

Antimicrobial stewardship programs should also support prospective audit and feedback, prescribing review, locally adapted clinical pathways, and collaboration among infectious-disease specialists, microbiologists, pharmacists, infection-control teams, emergency physicians, pediatricians, and primary care clinicians. Monitoring antibiotic use, treatment outcomes, CDI, adverse events, and resistance trends allows institutions to identify inappropriate practices and evaluate the effects of stewardship interventions [2,10,47,58].

Education is essential at both the professional and public levels. Clinicians require regular updates on treatment guidelines, diagnostic-test interpretation, and evolving local and international resistance patterns. Patients should receive clear explanations that antibiotics do not benefit most self-limiting diarrheal illnesses and may cause adverse reactions, disrupt the intestinal microbiota, promote CDI, and facilitate the acquisition of resistant organisms. Clear safety-net instructions—including when to seek reassessment for persistent symptoms, bloody diarrhea, dehydration, fever, or clinical deterioration—can reduce unnecessary prescribing without compromising patient safety [2,10,47,58,59].

Ultimately, effective stewardship combines selective testing, accurate interpretation, timely treatment of patients who are likely to benefit, and active discontinuation or de-escalation when antibiotics are unnecessary. Integration of these principles with surveillance, infection prevention, vaccination, sanitation, and One Health policies is essential to preserve antimicrobial effectiveness while maintaining safe and effective care for gastrointestinal infections.

## 7. Evidence Gaps and Stewardship-Relevant Innovations

Only innovations with a direct and plausible effect on prescribing or recurrence prevention are considered here: rapid diagnostics and microbiota-based therapies for recurrent CDI. Vaccines and artificial intelligence systems were removed because the available discussion did not support a sufficiently direct, evidence-based link to current antibiotic decisions.

### 7.1. Rapid Diagnostics and Treatment Reassessment

Multiplex gastrointestinal panels shorten time to organism detection and can facilitate earlier isolation decisions or pathogen-directed therapy. Their effect on antibiotic use is less consistent. Most studies are single-center before-and-after or retrospective analyses in which testing is bundled with stewardship review, and reductions in time to targeted therapy or antibiotic days are not universal. Evidence for effects on length of stay, complications, readmission, or mortality remains limited and heterogeneous. Thus, faster detection should not be equated with proven clinical benefit.

Clinical value depends on testing the right patient and acting on the result. Highly sensitive assays may detect colonization, prolonged shedding, nonviable organisms, or multiple organisms of uncertain relevance. A positive result should not automatically trigger treatment; a negative result should prompt reassessment of empirical antibiotics when the clinical condition permits.

Most panels do not provide a complete susceptibility profile. Selected resistance genes may not predict phenotype, while the absence of a target does not guarantee susceptibility. Reflex culture remains important when therapy, outbreak investigation, public-health reporting, or surveillance requires an isolate. The clearest stewardship opportunity is therefore a defined diagnostic pathway linking test eligibility, result interpretation, culture, and a time-limited antibiotic review.

Rapid diagnostics can support earlier initiation, narrowing, or discontinuation, but only when paired with clinical criteria and stewardship action. Prospective comparative studies should measure antibiotic exposure and patient-centered outcomes, not diagnostic yield alone.

### 7.2. Microbiota-Based Recurrence Prevention

Microbiota-based therapy is relevant to this review because it can prevent further antibiotic-treated episodes of recurrent CDI after completion of active antibacterial therapy. This is a specific recurrence-prevention strategy, not a general replacement for antibiotics in acute gastrointestinal infection.

Conventional fecal microbiota transplantation is effective for appropriately selected patients with multiple CDI recurrences, but its regulatory classification and permitted use vary across jurisdictions. Depending on the country and product, donor-derived microbiota may be regulated as a medicinal or biological product, a substance of human origin or tissue-related intervention, or under a specific discretionary framework. These differences affect donor eligibility, manufacturing standards, traceability, informed consent, adverse-event reporting, and whether treatment may be provided outside specialist centers or clinical trials. Clinicians should therefore follow current national requirements and use appropriately authorized services or products [43,45,46].

Donor screening reduces but does not eliminate the risk of transmitting infection. Reported safety concerns include transmission of multidrug-resistant organisms, enteric pathogens, emerging infectious agents, and antimicrobial-resistance genes. Screening programs should incorporate an updated medical and exposure history, appropriate testing of blood and stool, traceability of donor material, and procedures for retaining samples and investigating adverse events. Patients should be informed that unknown or newly emerging pathogens may not be detected by existing screening protocols. Particular caution is required in immunocompromised or critically ill patients, in whom the consequences of transmission may be severe [43,45,46].

Pediatric evidence remains less extensive than adult evidence. FMT should be considered only after multiple recurrences or refractory disease, following recommended antimicrobial regimens and multidisciplinary assessment; it is not indicated solely because an initial episode is severe [43].

Standardized microbiota-based products are available in some jurisdictions to prevent recurrent CDI in adults after antibacterial treatment. They improve manufacturing consistency but do not treat active fulminant disease and should not delay recommended antibiotics or surgery [44,46,51].

Outside recurrent CDI, evidence is insufficient to support routine microbiota-based treatment for acute infectious diarrhea. Claims that such therapies broadly reduce antibiotic use remain investigational and require comparative trials with safety, recurrence, antibiotic-exposure, and resistance outcomes.

## 8. Conclusions

Most acute gastrointestinal infections should be managed with rehydration and supportive care rather than antibiotics. Treatment should be reserved for severe, invasive, or high-risk presentations and should be reassessed when organism identification and susceptibility results become available.

The strongest practical safeguards are to avoid antibiotics when STEC is possible, obtain culture and susceptibility testing when results will change care, use destination- and region-specific resistance data cautiously, and select the shortest effective course. Important uncertainties remain, particularly the optimal duration for uncomplicated non-typhoidal *Salmonella* bacteremia and the clinical impact of multiplex panels outside stewardship-supported pathways.

For CDI, fidaxomicin is preferred for an initial non-fulminant episode when feasible, oral vancomycin is an acceptable alternative, and metronidazole is reserved for situations in which both are unavailable. Tigecycline should be framed only as specialist-directed salvage therapy. Microbiota-based interventions are relevant primarily to prevention after recurrent CDI, not to routine treatment of acute enteric infection.

## Figures and Tables

**Table 1:** Recommended antibiotic therapy for *Salmonella* infections according to clinical presentation.

Pathogen	Indications for Antibiotics	First-Line Therapy	Alternatives	Duration
Non-typhoidal *Salmonella* [4,9,14]	Bacteremia; immunocompromised patients; infants < 3 months; elderly; severe disease or systemic toxicity	Adults: Fluoroquinolone (Ciprofloxacin 500 mg PO twice daily Levofloxacin: 500 mg once daily) or Ceftriaxone 1–2 g IV once daily. Children: Ceftriaxone 50–75 mg/kg/day IV (max 2 g/day); Azithromycin 10–20 mg/kg/day (max 1 g/day) when appropriate according to susceptibility.	Amoxicillin 80–100 mg/kg/day in 3 divided doses (max 3 g/day); Trimethoprim–sulfamethoxazole (TMP component 8–10 mg/kg/day in 2 divided doses, max TMP 320 mg/day), according to susceptibility.	Gastroenteritis when treatment is indicated: usually 3–7 days. Uncomplicated bacteremia with rapid response and no focal infection: 5–7 days may be sufficient; extend for immunocompromise, persistent bacteremia, or endovascular/deep infection
*Salmonella Typhi*/*Paratyphi* [4,9,14]	Antibiotic therapy always indicated (enteric fever)	Adults: Fluoroquinolone (according to susceptibility) or Ceftriaxone 2 g/day IV. Children: Ceftriaxone 75 mg/kg/day IV (max 2 g/day) or Azithromycin 20 mg/kg/day (max 1 g/day); Fluoroquinolones may be considered in selected cases according to susceptibility and national recommendation.	Chloramphenicol 50–75 mg/kg/day in 4 divided doses (where still recommended and susceptibility confirmed).	7–14 days (longer in immunocompromised patients or according to clinical response)
Chronic carrier of *Salmonella Typhi* [4,9]	Chronic fecal carriage	Adults: Ciprofloxacin 750 mg twice daily or Norfloxacin 400 mg twice daily.	No standardized pediatric regimen; management should be individualized with an infectious disease specialist according to susceptibility testing.	4–6 weeks

**Table 2:** Recommended antibiotic therapy for *Shigella* infections according to disease severity and patient characteristics.

Pathogen	Indications for Antibiotics	First-Line Therapy	Alternatives	Duration
*Shigella* [4,9,15,14,17]	Moderate-to-severe disease; dysentery; infants and children with severe disease; malnutrition; immunocompromised patients; sepsis; outbreak control or institutional settings	Adults: Ciprofloxacin 500 mg PO twice daily (or 750 mg once daily) or Azithromycin 500 mg PO once daily. Children: Azithromycin 10–12 mg/kg/day PO (max 500 mg/day) for 3 days or Ciprofloxacin 15 mg/kg/dose PO every 12 h (max 500 mg/dose), according to susceptibility and national recommendations.	Adults and children: Ceftriaxone 50–100 mg/kg/day IV (max 2 g/day) for severe disease or hospitalization; Pivmecillinam (where available and susceptible); Carbapenems (e.g., Ertapenem 15 mg/kg IV every 12 h, max 1 g/day) for suspected or confirmed XDR Shigella.	3 days (5–7 days for severe or invasive infections, according to clinical response)

**Table 3:** Indications and recommended antibiotic regimens for *Campylobacter jejuni* infection.

Pathogen	Indications for Antibiotics	First-Line Therapy	Alternatives	Duration
*Campylobacter jejuni* [4,9,14,19]	Severe disease (high fever, dysentery, severe abdominal pain, prolonged diarrhea, or systemic illness); pregnancy; immunocompromised patients, including HIV; elderly or patients at increased risk of invasive disease	Adults: Azithromycin 500 mg PO once daily for 3 days or 1 g PO as a single dose. Children: Azithromycin 10 mg/kg/day PO once daily (max 500 mg/day) for 3 days. Alternative macrolide in children: Erythromycin 40 mg/kg/day PO in 4 divided doses (max 2 g/day) for 5 days.	Ciprofloxacin only when susceptibility is documented or reliable local resistance is low. For invasive or extraintestinal disease, select therapy with infectious-disease consultation according to species, infection site, host factors, and isolate susceptibility; aminoglycosides or carbapenems are not routine alternatives for enteritis.	Azithromycin: 1 dose or 3 days, according to regimen. Erythromycin: 5 days. Longer treatment may be required for invasive or extraintestinal disease.

**Table 4:** Recommended antimicrobial therapy for enterotoxigenic (ETEC) and enterohemorrhagic/Shiga toxin-producing (EHEC/STEC) *Escherichia coli* infections according to disease severity.

Pathogen	Indications for Antibiotics	First-Line Therapy	Alternatives	Duration
Enterotoxigenic *Escherichia coli* (ETEC) and other non-invasive bacterial travelers’ diarrhea [4,7,11,12]	Moderate diarrhea (interferes with planned activities); Severe diarrhea, including febrile diarrhea or dysentery	Adults: Azithromycin 1 g PO single dose or 500 mg PO once daily for 3 days. Children: Azithromycin 10 mg/kg/day PO once daily (max 500 mg/day) for 3 days. Adjunctive therapy: Loperamide may be considered in adults with moderate-to-severe non-dysenteric diarrhea.	Moderate, non-invasive diarrhea: Rifaximin 200 mg PO three times daily for 3 days (adults only). Fluoroquinolones (e.g., Ciprofloxacin): Adults 750 mg PO single dose or 500 mg PO twice daily for 3 days; Children: generally not recommended, but may be considered in selected cases according to susceptibility and national recommendations.	Single dose or 3 days, according to regimen and clinical response
Shiga toxin-producing *Escherichia coli* (STEC), including enterohemorrhagic *E. coli* (EHEC) [5,6,7]	Antibiotics are contraindicated	Supportive care only, including oral or intravenous rehydration. Renal function should be closely monitored and dialysis instituted when indicated.	No antimicrobial therapy recommended. Antimotility agents (e.g., loperamide) should also be avoided because of the increased risk of hemolytic uremic syndrome (HUS).	Supportive management only

**Table 5:** Clinical indications and recommended antibiotic regimens for *Vibrio cholerae* infection.

Pathogen	Indications for Antibiotics	First-Line Antibiotics	Alternatives	Duration
*Vibrio cholerae* [7,9,25,26,27,28,29,30,31]	Moderate-to-severe dehydration; severe cholera; profuse watery diarrhea after rehydration; selected immunocompromised patients or severely ill infants with strong clinical suspicion	Adults: Doxycycline 300 mg PO single dose. Children: Doxycycline 6 mg/kg PO single dose (where appropriate according to age and national recommendations). Alternative first-line: Azithromycin 1 g PO single dose (adults) or 20 mg/kg PO single dose (max 1 g) in children.	Adults: Ciprofloxacin 1 g PO single dose or 500 mg PO twice daily for 3 days (according to susceptibility). Children: Ciprofloxacin 15 mg/kg/dose PO every 12 h (max 500 mg/dose) only when indicated. Ceftriaxone may be considered for severe infection when oral therapy is not feasible. Tetracycline: Adults 500 mg PO four times daily for 3 days; Children 12.5 mg/kg/dose PO four times daily for 3 days (where appropriate)	Single dose (preferred) or 3-day course according to the selected regimen and clinical response.

**Table 6:** Clinical indications and recommended antimicrobial regimens for *Clostridioides difficile* infection (CDI). Pediatric fidaxomicin doses are per administration every 12 h for 10 days and should be checked against current jurisdiction-specific product information.

Pathogen	Indications for Antibiotics	First-Line Antibiotics	Alternatives	Duration
Initial non-fulminant CDI [34,35,41,42]	Symptomatic CDI confirmed by stool toxin assay or compatible microbiological testing	Adults: fidaxomicin 200 mg PO every 12 h for 10 days. Pediatric oral suspension every 12 h for 10 days: <4 kg, 40 mg; 4 to <7 kg, 80 mg; 7 to <9 kg, 120 mg; 9 to <12.5 kg, 160 mg; ≥12.5 kg, 200 mg. Use the approved formulation and jurisdiction-specific product information	Vancomycin: adults 125 mg PO four times daily; children 10 mg/kg/dose PO four times daily (max 125 mg/dose). Metronidazole only for an initial non-severe episode when both fidaxomicin and oral vancomycin are unavailable; it is not a preferred equivalent option.	10 days
Severe, non-fulminant CDI [34,35,42]	Leukocytosis or increased serum creatinine without hypotension, shock, ileus, or toxic megacolon	Fidaxomicin 200 mg PO every 12 h or vancomycin 125 mg PO four times daily	Use the standard non-fulminant regimen; high-dose vancomycin plus intravenous metronidazole is not indicated solely for severe laboratory criteria.	10 days
Fulminant CDI [34,35,42]	Hypotension or shock, ileus, toxic megacolon, or other critical illness	Vancomycin 500 mg PO or by nasogastric tube four times daily plus metronidazole 500 mg IV every 8 h	Add rectal vancomycin when ileus is present. Tigecycline is non-standard salvage therapy only after specialist consultation; do not delay surgery.	Approximately 10 days; individualize to response and source-control needs
Recurrent CDI [34,35,42,43,44,45,46]	Recurrence within 8 weeks after resolution of a previous episode	Preferred: fidaxomicin 200 mg PO twice daily for 10 days or an extended-pulsed regimen. A vancomycin taper-and-pulse regimen is an alternative	Bezlotoxumab as an adjunct in selected high-risk patients; fecal microbiota transplantation or an approved microbiota-based product after multiple recurrences; vancomycin followed by rifaximin in selected patients.	Fidaxomicin: 10 days or extended-pulsed regimen. Vancomycin taper: regimen-dependent, often several weeks

## Data Availability

No new data were created or analyzed in this study. Data sharing is not applicable to this article.
